# Consequence of alcohol intoxication-mediated efferocytosis impairment

**DOI:** 10.3389/fimmu.2024.1386658

**Published:** 2024-07-22

**Authors:** Subhashini Brahadeeswaran, Ramasamy Tamizhselvi

**Affiliations:** School of Biosciences and Technology, Vellore Institute of Technology, Vellore, Tamil Nadu, India

**Keywords:** alcohol, apoptotic bodies, efferocytosis, inflammation, ROS, secondary necrosis

## Abstract

Alcohol ingestion is a widespread habituation that evolved along with a growing population, altering physiological conditions through immunomodulatory function. There is much research that has reported that consumption of alcohol at low and heavy levels causes different biological impacts, including cellular injury, leading to systemic dysfunction and increased inflammatory markers. In the fate of professional phagocytic cells, efferocytosis is an inevitable mechanism activated by the apoptotic cells, thus eliminating them and preventing the accumulation of cell corpses/debris in the microenvironment. Subsequently, it promotes the tissue repair mechanism and maintains cellular homeostasis. Unfortunately, defective efferocytosis is widely found in several inflammatory and age-related diseases such as atherosclerosis, autoimmune diseases, lung injury, fatty liver disease, and neurodegenerative diseases. Alcohol abuse is one of the factors that provoke an immune response that increases the rate of morbidity and mortality in parallel in systemic disease patients. Information regarding the emergence of immunomodulation during alcoholic pathogenesis and its association with efferocytosis impairment remain elusive. Hence, here in this review, we discussed the mechanism of efferocytosis, the role of defective efferocytosis in inflammatory diseases, and the role of alcohol on efferocytosis impairment.

## Introduction

Drinking alcohol is prevalent among people of different age brackets in this modern era (1). Alcohol consumption can induce more than 200 pathologies and morbidities, as per a World Health Organization (WHO) report (2). The associated conditions can be detrimental and severe. Alcohol can elicit inflammation and impair multiple organs by stimulating the innate immune system, elevating the likelihood of premature mortality and socioeconomic burden (3). Alcohol is an exogenous toxin that can perturb intestinal homeostasis and modulate the gut microbiota, leading to the secretion of endotoxins such as lipopolysaccharide (LPS) into the liver through the portal vein (4). *In-vitro* and *in-vivo* studies have depicted that the alcohol-induced inflammatory signaling pathway, via the upregulation of ethanolic metabolites, promotes reactive oxygen species (ROS) production. Excessive ROS is one of the hallmark triggering factors that induce the transcriptional activation of the nuclear factor kappa-light-chain-enhancer of activated B cells (NF-κB) pathway which further aids in accelerating pro-inflammatory cytokine secretion. This can result in alcoholic liver diseases, such as steatosis, hepatitis, and cirrhosis (5). In addition, alcohol can readily traverse the blood-brain barrier and access the central nervous system, injuring the neurons and transiently affecting cognitive and locomotor functions (6). Moreover, numerous reports have indicated that long-term alcohol exposure predisposes patients to other disease conditions such as lung infections, cardiomyopathy, and stroke (7, 8). Under the aforementioned unfavorable conditions, tissue-resident macrophages become activated and recruit more leukocytes to the lesion site. These immune cells produce a plethora of immune effectors that facilitate apoptosis (9).

**Figure 1:**
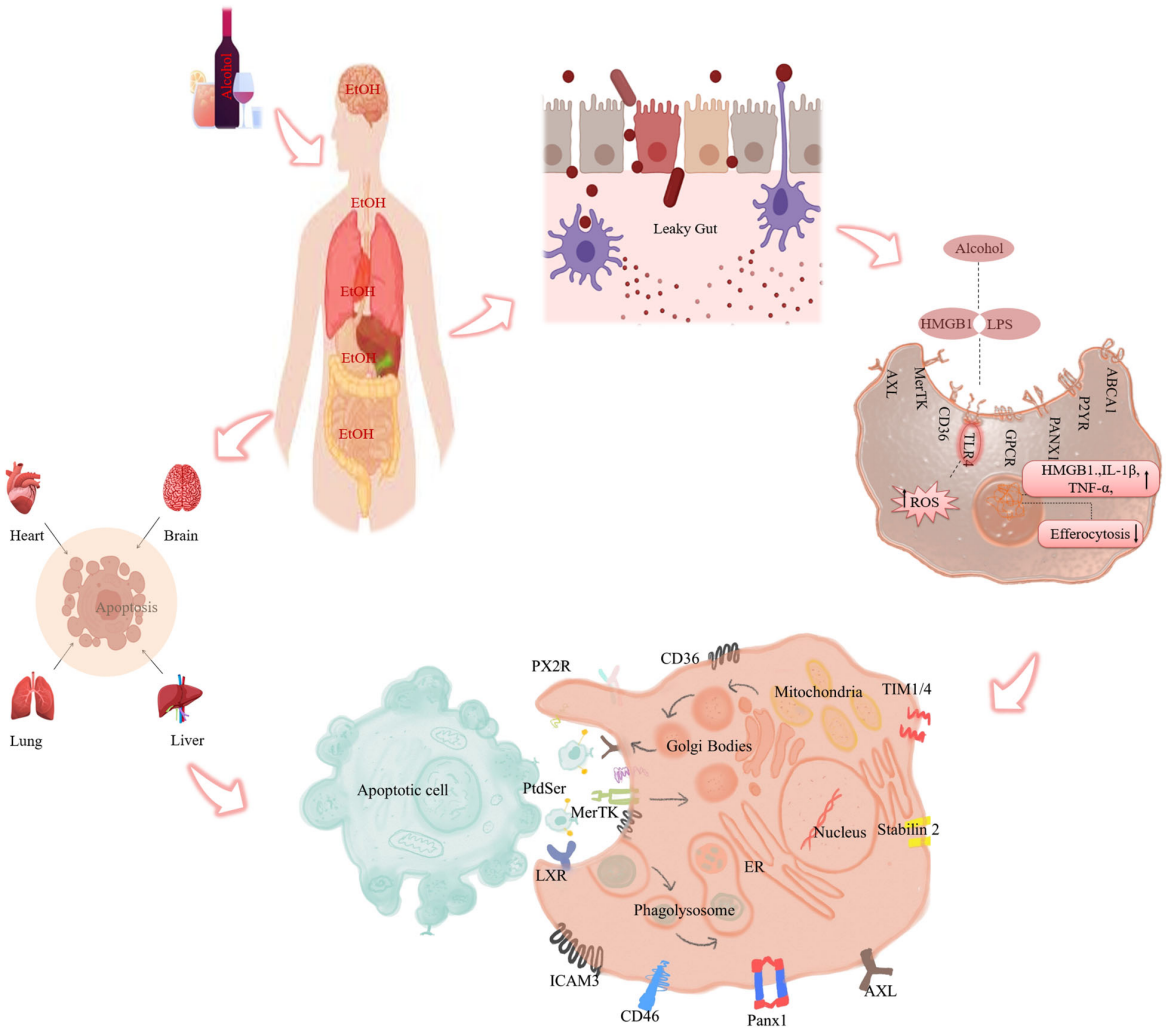
Role of alcohol mediated efferocytosis impairment and inflammation. Alcohol ingestion causes intestinal permeability and allows endotoxins (LPS) to leak into the portal circulation and reach various organs such as the heart, lungs, liver, and brain. There, LPS activates phagocytes and induces the release of pro-inflammatory cytokines via ROS production. At the same time, it impairs the intracellular signaling pathways involved in efferocytosis. (In detail: Ethanol-induced impairment of efferocytosis) Defective efferocytosis leads to the accumulation of apoptotic cells in the tissue, which triggers secondary necrosis and inflammation.

*Apoptosis* is a tightly regulated cellular mechanism that mediates the programmed cell death of damaged or senescent cells to preserve cellular sterility (10). Efferocytosis is the phagocytic clearance of apoptotic cells (ACs) by specialized and non-specialized phagocytes (11). Efferocytosis prevents the secondary necrosis of apoptotic cells and the release of their intracellular components, which could impair tissue homeostasis (12). Chronic alcohol exposure can induce oxidative stress and inflammation, increasing the apoptotic cell load that needs to be removed by efferocytosis and causing a heavy burden (13). However, alcohol can also impair efferocyte function and compromise the clearance of apoptotic bodies, which may contain damage-associated molecular patterns (DAMPs) that induce cell necrosis (14). Thus, the impact of alcohol on the efferocytosis process is unclear. This review will discuss how alcohol affects efferocytosis and potential therapeutic interventions (**Figure 1**).

## Multistage process of efferocytosis function in the efficient elimination of cell corpses

The efferocytosis process involves removing apoptotic cells by various types of phagocytes, such as neutrophils, macrophages, dendritic cells, fibroblasts, epithelial cells, and other immune cells (15). Efferocytosis consists of four sequential steps: recruitment, recognition, internalization, and digestion of the apoptotic cells. In healthy conditions, efferocytosis efficiently clears the millions of apoptotic cells generated daily, preventing their accumulation and potential immunogenicity. Four types of signals mediate efferocytosis: ‘find me’ signals (eg. sphingosine-1-phosphate (S1P), lysophosphatidylcholine (LPC), C-X-C motif chemokine ligand 1 (CX3CL1), and nucleotides) that attract phagocytes to the site of apoptosis, ‘eat me’ signals (eg. Phosphatidylserine, (PS)) that mark the apoptotic cells for phagocytosis, ‘don’t eat me’ signals (eg. lactoferrin glycoprotein, (CD36)) that inhibit the uptake of viable cells, and bridging molecules (growth arrest-specific 6(GAS6)) that facilitate the interaction between the apoptotic cells and the phagocytes (16). However, alcohol intoxication impairing the efferocytosis mechanism enhances adverse inflammatory diseases.

## Phagocyte recruitment and recognition of apoptotic cells

Once the cell undergoes senescence or PAMP/DAMP-mediated cell death the adjacent phagocytes are more prone to remove those cells from that locale via the secretion of various bioactive molecules from the dying cell. A few molecules, such as adenosine triphosphate (ATP), uridine triphosphate (UTP), S1P, LPC, CX3CL1, calreticulin, heparin, pentaxin3, high mobility group box 1 (HMGB1), and S100 are called ‘find me’ signals or chemotactic factors (17–19). They are generated by the activation of pro-apoptotic proteins and caspases that execute the apoptotic pathway. The ‘find me’ signals interact with specific receptors on the membrane of adjacent phagocytes, such as macrophages and dendritic cells. These receptors comprise G-protein-coupled receptor (GPCR), S1P receptor (S1PR), ATP-binding cassette transporter A1 (ABCA1), P2Y receptors (P2YRs), and pannexin-1 (PANX1) (20). The interaction of the ‘find me’ signals induces the chemotaxis of the phagocytes to the location of the apoptotic cell. A lack of any receptors could lead to the accumulation of apoptotic cells that inhibit the subsequent stages involved in the efferocytosis process.

## Recognition of apoptotic cells

During apoptosis, the expression of scramblase increases to move the membrane-bound phosphatidyl serine from the inner to the outer region of the plasma membrane with a decrease in the activity of flippase. Flippase usually keeps phosphatidyl serine (PtdSer) inside the membrane of living cells instead of exposing it as a hallmark signal for phagocytosis. Apoptotic cells project the phosphatidyl serine on their surface, which attracts phagocytes through various receptors, such as TAM receptors [TYRO3 protein tyrosine kinase (TYRO3)], tyrosine-protein kinase receptor UFO (AXL), c-mer proto-oncogene tyrosine kinase (MERTK), T cell immunoglobulin and mucin domain-containing protein 3 (TIM4), Brain angiogenesis inhibitor 1 (BAI1), TIM3, the cluster of differentiation (CD) 300 family, STABILIN 1 and 2, a receptor for advanced glycation end product (RAGE), scavenging receptor class B members (CD36), and a opsonizing factor C1q (20). Another protein on apoptotic cells, calreticulin, also helps phagocytosis by sending an ‘eat me’ signal. In addition, some soluble proteins act as bridges between apoptotic cells and phagocytes, such as growth arrest-specific protein 6 (GAS6), protein S (PS) gene (ProS1), milk fat globule-EGF factor 8 (MFG-E8), and developmental endothelial locus-1 (DEL-1). These proteins bind to apoptotic and phagocytic receptors through their N and C terminal domains. Likewise, apoptotic cells are engulfed in the efferocytosis process.

## Internalization of the apoptotic bodies

The engulfment process involves the recognition and binding of the ‘eat me’ signal on the apoptotic cell surface, activating the intracellular signaling cascade to form a phagocytic cup by altering the cytoskeleton during efferocytosis. The phagocytic cells extend their cell membrane as lamellipodia to surround and internalize the apoptotic bodies, forming a phagosome. Rac, a member of the Ras homolog family (Rho) GTPase family, is essential for the shape change and engulfment of the apoptotic cell. The activation of Rac is mediated by different receptors that bind to the ‘eat me’ signal, such as tyrosine kinase cell surface transmembrane receptors (TAM), BAI1, low-density lipoprotein-receptor–related protein (LRP1), and stabilin2, and their downstream effectors, such as crkII, dedicator of cytokinesis (dock)180, engulfment and cell motility 1 (ELMO1), and GULP (21). The cell movement and cytoskeletal rearrangement required for the uptake of apoptotic cells depend on the upstream regulators of Ras-related C3 botulinum toxin substrate 1 (Rac1), such as crkII, dock180, and ELMO1 (21). Furthermore, other Rho GTPase family members, such as RhoA and cell division cycle (cdc42), regulate cytoskeletal rearrangement and actin polymerization on the phagocyte membrane site, which is strongly influenced by the activation/inhibition of Rac1, RhoA and Rho-associated coiled-coil-containing protein kinase (ROCK), respectively. Reports suggested that overexpression of RhoA impairs AC engulfment through activation of ROCK (22). ROCK kinase-activated myosin light chain phosphorylation facilitates actomyosin accumulation and cellular contraction to enclose apoptotic bodies during the process of engulfment.

In addition, Rho GTPase members can switch on or off the efferocytosis mechanism by binding with GTP or GDP. Rho GTPase activator, a guanine nucleotide exchange factor (GEF), is one of the leading factors that increase the activity of Rac1 by regulating GDP and allowing GTP to bind to Rho family members (23). After ingestion, Rac1 activity in the phagocytic membrane decreases, along with F-actin disassembly and phagocytic cup closure. Also, RhoA expression was increased before phagocytosis rather than after engulfment. This is necessary for the effective action of AC engulfment and cell migration in mammalian efferocytosis. However, if the activation of these dynamic proteins is disrupted, it can lead to uncontrolled engulfment, such as rapid and indiscriminate eating.

## Phagolysosome fusion mediated degradation and post-engulfment of AC

Once phagocytes engulf the apoptotic cells it subsequently forms phagosomes that merge with lysosomes to break down the phagosome contents. This process requires specific GTPase proteins, such as Ras-associated binding (RAB) 5 and RAB7, which regulate the maturation of early and late phagosomes. Blocking RAB7 prevents the fusion of phagolysosomes. Moreover, soluble-N-ethylmaleimide-sensitive-factor accessory-protein receptor (SNARE) proteins, such as vesicle-associated membrane protein (VAMP) 7 and syntax 7, facilitate the formation of phagolysosomes (24). Other proteins, such as RILP, dynein, lysosome-associated membrane protein 1 (LAMP) 1, and LAMP2, are also essential for this process. In addition, autophagy-related proteins can attach microtubule-associated protein 1A/1B-light chain (LC) 3 proteins to the phagosome membrane, forming LC3-associated phagocytosis (LAP). LAP enhances the degradation of apoptotic cell components by promoting the fusion of phagosomes and lysosomes (24). Impairment of LC3 attachment delays the acidification and degradation of phagosomes. After the fusion of phagolysosomes, phagocytes break down the engulfed macromolecules which benefits lysosomal enzymes such as nucleases, proteases, lipases, sulfatases, other phospholipases, phosphatases, and acid hydrolases. The pH of lysosomes changes to enable their fusion with suitable endosomes or phagosomes. The phagocytic process is impaired by the lack of fusion between the phagosome and the lysosome, resulting in the persistence of the apoptotic cell-derived material within the phagocyte.

## Internalized factor promoting continual efferocytosis

Engulfed contents, such as sterols, act as ligands for nuclear receptors that modulate gene expression in response to metabolic cues. Even the glycolysis pathway is involved in the efficient removal of the apoptotic cell from the vicinity through the continuous secondary activation of efferocytosis. Efferocytosis also modulates the anti-inflammatory response by interacting with nuclear receptors such as peroxisome proliferator-activated receptor gamma (PPAR-γ) and liver X receptors (LXR-α) heterodimer with retinoid X receptor (RXR) α/β that bind to the lipid derivatives of apoptotic cell (25). Thus, this leads to an increase in the expression of genes that necessitate the clearance of apoptotic cells, such as MerTK, CD36, C1q, and MFG-E8, which mediate the recognition and binding of apoptotic cell surface markers. Furthermore, these receptors enhance the production of anti-inflammatory cytokines such as interleukin (IL) 10 and tumor growth factor (TGF) beta (26). Moreover, efferocytosis stimulates the synthesis of pro-resolving mediators such as resolvin D1 (RvD1), lipoxin A4 (LXA4), and prostaglandin E2 (PG2E) that promote the resolution of inflammation (27).

In addition, Park et al. demonstrated that uncoupling protein (UCP) 2 deficiency in phagocytes impairs the coupling of energy production and nutrient metabolism of apoptotic cells, resulting in decreased efferocytosis capacity. UCP2 is a proton carrier that dissipates the proton gradient across the inner mitochondrial membrane, lowering the mitochondrial membrane potential without generating ATP. Thus, UCP2 modulates the clearance efficiency of phagocytes by regulating the mitochondrial membrane potential. These findings suggest that there may be interactions and interconnections among the different stages of efferocytosis. However, testing and validating these hypotheses remains a challenge.

## “Don’t eat me” signal or anti-efferocytosis signal

**Figure 2:**
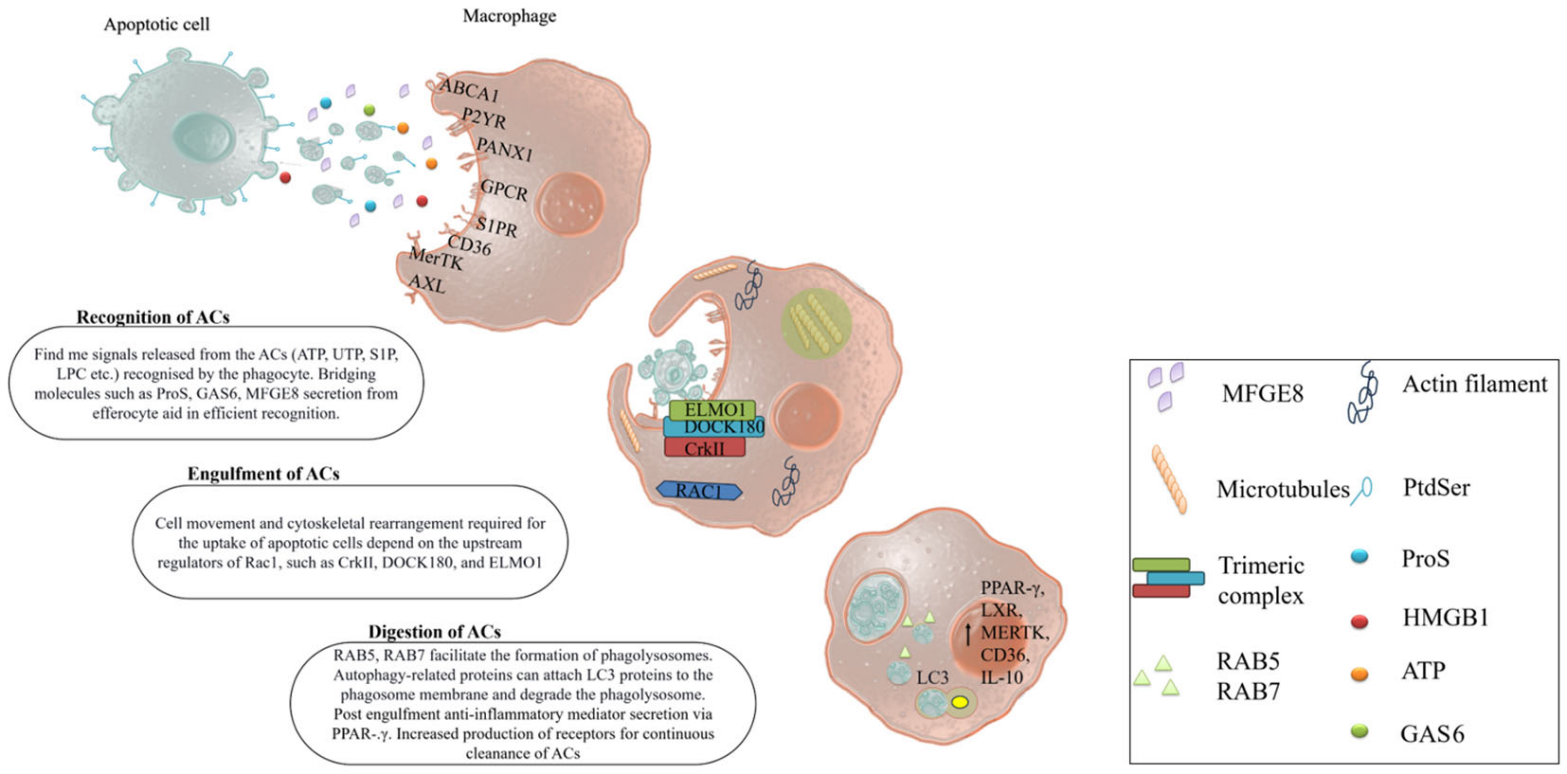
Mechanism of efferocytosis. Apoptotic cells are cleared by professional phagocytes (macrophages, dendritic cells, neutrophils). They quickly sense the ‘find me’ signals from the apoptotic cells via their prompt receptors. Some bridging molecules also help them recognize and activate efferocytosis by linking to both apoptotic cells and phagocytes. The phagocytes engulf the apoptotic cells through cell rearrangement gene expression. The fusion of phagosome and lysosome degrades the engulfed apoptotic cells. The degradation products activate signaling pathways that boost efferocytosis by increasing the expression of related genes.

The efferocytosis mechanism specifically produces anti-inflammatory cytokines that change the phagocyte phenotype to resolve inflammation. Preventing the release of DAMPs and pro-inflammatory cytokines can reduce the severity of many diseases. Classically, healthy cells express ‘don’t eat me’ signals or ‘keep me’ signals such as CD47, the specific marker of anti-efferocytosis. CD47 binds to SIRP-α on phagocytes and inhibits the uptake of apoptotic cells. However, apoptotic cells reduce the expression of CD47 compared to healthy viable cells. Similarly, other ‘don’t eat me’ signal mediators such as CD300a, CD31, and major histocompatibility complex (MHC) interact with their cognate receptors such as CD300a, CD31, and LILRB1 respectively, thus regulating the recognition of apoptotic cells and block the efferocytosis mechanism. The rapid and effective clearance of apoptotic cells is essential for preventing secondary necrosis, as well as for tissue repair and inflammation resolution (**Figure 2**).

## Defective efferocytosis mediated systemic and inflammatory diseases

Efferocytosis, the swift engulfment and removal of apoptotic cells by efferocytes, is an essential cellular process that underpins immune tolerance and mitigates disease progression. Deficiencies in this clearance mechanism can precipitate a persistent inflammatory state and the generation of autoantigens, culminating in the onset of autoimmune disorders and life-threatening conditions. These include acute pulmonary and hepatic injuries, diabetes, cardiovascular and cerebrovascular events, and neurodegenerative diseases, all of which have been associated with impaired efferocytotic activity.

Accumulated studies suggest that chronic alcoholic exposure significantly leads to type 2 diabetes mellitus (T2DM). Glucose metabolism is the one required for the phagocytes to continuously engulf the apoptotic bodies during the process of efferocytosis by gaining energy. Glucose imbalance in the metabolic activity was observed in the T2DM and showed that efferocytosis impairments were associated with a cellular deformity such as pancreatic islet of beta cells, adipose tissue, and skeletal muscle dysfunction (28). Previous reports suggested that efferocytosis impairment occurred via the production of ROS and ER stress enabling the M1 phenotypic macrophage polarization. A lack of membrane-bound receptors in the efferocyte elevated the efferocytosis impairment through the overexpression of ADAM metallopeptidase domain 17 (ADAM-17) activation. As a result, the ADAM17-mediated cleavage of membrane-bound receptor MerTK facilitates secondary necrosis and causes the formation of plaque in atherosclerosis (29). A recent study reported that downregulation of LRP-1 showed pro-inflammatory cytokines and chemokines such as tumor necrosis factor (TNF-α), MMP-9, and MCP-1 thus further enhancing the recruitment of immune cells that led to sterile inflammation (30). Yang et al. reported a novel mechanism of epigenetic regulation that affects defective apoptotic signaling and efferocytosis in chronic pancreatitis (CP). They showed that Atp8b1, a flippase protein that translocates LPC to the outer leaflet of the plasma membrane, is downregulated in the acinar cells of *PRSS1* ^Tg^ CP mice. LPC is a ‘find me’ signal that facilitates the recognition and clearance of apoptotic cells by macrophages. Reduced Atp8b1 expression leads to impaired LPC exposure and accumulation of apoptotic acinar cells, which triggers inflammation and fibrosis. By integrating ATAC-seq and RNA-seq data, they identified Bhlha15 as a key transcription factor that regulates Atp8b1 expression and phospholipid metabolism in acinar cells (31). Thus, this study reveals a novel epigenetic pathway that modulates efferocytosis and its implications for CP pathogenesis and therapy. Even respiratory diseases such as chronic obstructive pulmonary disease (COPD), pneumonia infection, acute lung injury, and acute respiratory distress syndrome (ARDS) were characterized by increased immune response and altered efferocytosis pathway. Specifically, the regulation of TAM receptors such as Axl causes greater susceptibility to inflammatory situations in the respiratory tract, ultimately enhancing acute lung injury (ALI) and other airway-mediated diseases (32). Also, other efferocytic genes such as MFGE8, MerTK, and PtdSer were downregulated in both the tissue-resident macrophage, alveolar macrophage and apoptotic cells respectively (33). In their 2020 study, Shibata and colleagues observed an upregulation of GAS6, a bridging protein in macrophages mediated by the activation of Signal transducer and activator of transcription (STAT) 6 signaling, which corresponded with a reduction in acute ALI (34). Furthermore, the authors identified excessive NETosis as a pivotal cellular process contributing to the development of ALI. Therefore, they demonstrated that inhibition of HMGB1 in bronchoalveolar lavage fluid reinstated AMPK signaling, thereby enhancing the efferocytosis rate directed at neutrophil extracellular traps (NETs) (34). Interestingly, the expression of the ‘don’t eat me’ signal CD47 was depleted in COPD and was reversed during corticosteroid treatment (35). Altogether this indicates that impaired efferocytosis could be a potent pathological mediator for systemic disorders.

In the context of autoimmune disorders, the efferocytosis mechanism is initiated by the identification of self-antigens released during apoptosis or necrosis. Research indicates that disruptions in the intricate efferocytosis pathway can precipitate conditions such as systemic lupus erythematosus (SLE) and rheumatoid arthritis (RA) (36). Investigations into SLE have demonstrated a propensity for patients to accumulate apoptotic cells within the germinal centers of lymph nodes (37). Notably, Hanayama et al. discovered that mice lacking the protein MFG-E8 exhibited compromised C1q complement function, leading to excessive accumulation of dead cells and the production of autoantibodies (38). Similarly, RA is a systemic ailment marked by increased rheumatoid factor and a persistent immune response localized to the synovial joint regions. The prompt activation of efferocytosis is essential in the synovial tissue to mitigate the generation of secondary necrosis (39). However, research has identified that a deficiency in DNaseII impedes the degradation of DNA from apoptotic cells, thereby obstructing efficient clearance of dead cells and contributing to the elevation of pro-inflammatory cytokines such as TNF-α, IL-1β, and IL-6, which exacerbate RA (40, 41). Analysis of synovial fluid and tissue from patients with RA has demonstrated a marked elevation in the expression levels of the soluble TAM receptor tyrosine kinases Tyro3, Axl, and MerTK. This upregulation is attributed to the proteolytic activity of the sheddase ADAM17. The resultant cleavage of TAM receptors is implicated in the disruption of apoptotic cell clearance (42, 43). Presently, the generation of soluble TAM receptors (sTAM) is recognized as a potential biomarker for the detection of efferocytosis dysfunction and its related pathologies. In parallel, clinical investigations have disclosed that the persistence of non-phagocytosed apoptotic cells is indicative of impaired efferocytosis in osteoarthritis patients (42, 44). SLE is characterized as a chronic, multisystemic condition that modulates the immune system and hinders efferocytosis. Under normal physiological conditions, C1q serves as an opsonin that adheres to apoptotic cells (ACs), thereby preserving homeostasis through the facilitation of efferocytosis (45). Contrastingly, C1q-deficient mice exhibited a marked accumulation of apoptotic cells, which is implicated in the onset of SLE (36). These findings corroborate the hypothesis that defective efferocytosis may interrupt self-tolerance and play a role in the etiology of various autoimmune diseases. Nonetheless, additional research is imperative to elucidate the mechanisms involved. In the context of cardiac dysfunction, mice lacking MerTK and MFG-E8 displayed an enlargement of infarct size, compromised cardiac functionality, and a decrement in myocardial repair capabilities. The therapeutic administration of MerTK, C1q, MFG-E8, and neutrophil apoptotic bodies has been shown to significantly mitigate myocardial infarction by rectifying impaired efferocytosis (46–48). Furthermore, the application of a neutralizing anti-CD47 antibody has been observed to alleviate the inhibitory signals, thereby enhancing efferocytosis in ischemic myocardial reperfusion (49). These studies underscore a profound link between efferocytosis and the resolution of inflammation in cardiac anomalies, highlighting the therapeutic potential of modulating efferocytosis to reduce myocardial ischemia-reperfusion injury and modulate the immune response.

## Exposure to alcohol-mediated efferocytosis impairment

Efferocytosis, the process of clearing apoptotic cells, is impaired by several factors, including systemic alcohol exposure. It is well-established that the liver serves as the primary organ for alcohol detoxification, utilizing a trio of enzymes, namely, alcohol dehydrogenase (ADH), cytochrome P450 2E1 (CYP2E1), and catalase (50). Excessive alcohol intake precipitates the accumulation of protein adducts and ROS, culminating in damage to both hepatic resident cells and infiltrating macrophages (51). This damage is exacerbated by alcohol-induced necrosis, which elevates pro-inflammatory cytokine secretion locally, thereby intensifying the immune response. Despite the occurrence of apoptosis, a deficiency in efferocytosis has been observed within liver-specific cells, including hepatocytes and Kupffer cells (52). Prior studies have indicated that alcohol consumption adversely affects efferocytosis rates, leading to an increase in secondary necrosis, irreversible cellular damage, and an amplified immune response within the liver (52). Notably, the sinusoidal surface of hepatocytes, particularly the asialoglycoprotein receptor (ASGP-R), plays a crucial role in the recognition of apoptotic cells within the liver. Intriguingly, research conducted by MC Vicker and colleagues demonstrated that ethanol-fed rats exhibited a reduced phagocytosis rate compared to controls. Furthermore, chronic alcohol exposure in rats deficient in ASGPR resulted in diminished clearance of apoptotic cells. The effect of ethanol on asialoglycoprotein receptor-mediated phagocytosis of apoptotic cells was diminished in rat hepatocytes (53). These findings suggest that impaired phagocytosis contributes to secondary necrotic cell death, which in turn may trigger systemic inflammation.

Recent studies have consistently indicated that alcohol-related liver diseases, such as hepatitis, steatosis, and fibrosis, are precursors to chronic inflammation and subsequent systemic organ damage (54). Consequently, to mitigate the acceleration of cell death induced by alcohol, research efforts are being directed toward elucidating the mechanisms of efferocytosis and its implications across various tissue-specific sites. Intriguingly, emerging research is delineating the nexus between efferocytosis and chronic inflammatory diseases. The investigation led by Hardesty et al. has highlighted the pivotal role of pro-resolving mediators released during efferocytosis in forestalling necrotic cell death and systemic dysfunctions. Their findings advocate that in alcohol and LPS-induced murine models, administration of resolvin D1 (RvD1) markedly attenuates neutrophil infiltration and the secretion of pro-inflammatory cytokines within the liver. Furthermore, RvD1 has been shown to inhibit the pyroptotic pathway by downregulating genes associated with inflammasome activation, such as caspase-11 and gasdermin-D (55). Collectively, the prompt clearance of apoptotic cells may diminish the activation of ancillary inflammatory signaling pathways, thereby facilitating the resolution of inflammation.

Contemporary research indicates that neutrophils are predisposed to instigate the formation of neutrophil extracellular traps (NETs), which are notably augmented in cases of alcoholic liver diseases. The process of NETosis, precipitated by NET formation, necessitates expeditious phagocytosis by macrophages to mitigate the intensification of such liver conditions. For example, an upsurge in NET accumulation has been observed in acute sepsis induced by excessive alcohol consumption (56). In parallel, experimental models involving mice subjected to binge alcohol intake followed by phorbol myristate acetate (PMA) administration exhibited an initial decline in NET production (56). During the efferocytosis phase, an increase in citrullinated histone H3-a marker for NETs was detected, leading to a decelerated clearance rate. Significantly, the abatement of NETs was correlated with amelioration of exacerbated hepatic inflammation. However, it is evident that alcohol consumption detrimentally affects both neutrophils and macrophages, culminating in a diminished engulfment of NET-containing neutrophils and a surge in pro-inflammatory cytokines that contribute to hepatocyte necrosis (52). Consequently, prompt phagocytic removal of these elements is imperative to prevent necrotic cell death and subsequent tissue damage.

The investigation posits that both acute and chronic exposure to alcohol initiates a cascade of genetic responses that modulate efferocytic pathways, specifically through the downregulation of MFG-E8 and the upregulation of HMGB1. Notably, these genetic expressions were significantly modified in RAW.264.7 macrophages and Kupffer cells that were subjected to alcohol treatment, in stark contrast to their unaltered counterparts in control groups. In parallel, an elevation in HMGB1 levels was observed in the serum of mice with chronic alcohol induction (57). Collectively, these findings implicate the impairment of efferocytosis by alcohol as a pivotal factor exacerbating alcohol-related hepatic pathologies and other systemic inflammatory conditions. Furthermore, it underscores the necessity for meticulous research to ascertain whether alcohol exerts a direct inhibitory effect on the efferocytosis mechanism. The current landscape of chronic inflammatory diseases is riddled with questions that remain unanswered, yet the advancement of efferocytosis research holds the promise of elucidating these mysteries in the foreseeable future.

Neurodegeneration is a term that encompasses the gradual deterioration of neuronal structure and function over time. One of the key processes that maintain neuronal health and prevent neuroinflammation is efferocytosis, which is the clearance of apoptotic neurons and amyloid-beta (Aβ) plaques from the brain. Alcohol intake can interfere with efferocytosis through various pathways, for example, by compromising the intestinal barrier, which facilitates the translocation of bacteria and bacterial endotoxins into the bloodstream and the brain, where they stimulate microglia and trigger neuro-inflammation. Reduced efferocytosis can result in the accumulation of ACs and the secretion of pro-inflammatory mediators, which can induce neuro-inflammation and neuro-degeneration. MFGE8 is a molecule that regulates the microglial phagocytosis of apoptotic neuronal cells and attenuates inflammation. MFGE8 can also inhibit A1 astrocytes and modulate microglia between M1/M2 by regulating the NF-κB and PI3K-AKT pathways to protect neurons from death. *In-vivo* studies showed a reduced level of MFGE8 expression in the Alzheimer’s disease model (58). In CNS, microglial phagocytosis is facilitated by bridging proteins such as MFGE8 and Pro S as TAM-receptor kinases such as Axl and MerTK (59). MFGE8 has also been demonstrated to prevent Parkinson’s disease by protecting mesencephalic dopamine neurons (60). In the alcohol-induced model, MFGE8 expression was impaired, which might be a significant mechanism of alcohol-mediated impaired efferocytosis leading to neurodegeneration. Alcohol consumption can impair efferocytosis by reducing the expression and activity of phagocytic receptors, such as CD36, MerTK, and Axl, on microglia, which are the main phagocytes in the brain. This effect can lead to the accumulation of apoptotic neurons and Aβ in the brain, which can trigger neuroinflammation and neurodegeneration. Alcohol exposure increases the levels in the brain of HMGB1, a nuclear protein that can be released by damaged or activated cells. This has been observed in both human alcoholics and animal models of alcoholism. HMGB1 impairs the clearance of apoptotic neutrophils by inhibiting the exposure of PS, a phospholipid that signals the efferocyte to engulf the dying cells (61). Moreover, HMGB1 interacts with TLR4, a receptor that triggers ROS generation and inflammatory responses, which further hampers efferocytosis.

A recent study on RhoA, a regulator of efferocytosis, showed that alcohol exposure combined with LPS treatment, but not alone, reduced efferocytosis in both *in vitro* and *in vivo* models. The study also found that ROCK activation was increased by alcohol exposure, but not RhoA. Blocking ROCK and RhoA revealed that the alcohol-induced impairment of alveolar macrophages was independent of RhoA, suggesting a key role of ROCK in efferocytosis dysfunction and the development of ARDS and pulmonary inflammation (13) Moreover, alcoholic subjects are more susceptible to bacterial infection due to impaired phagocytosis function, which was observed in alcoholic hepatitis, sepsis, and lung injury (62, 63). Phagocytosis and LAP require glycolysis as an energy source. Previous reports showed that acute alcohol exposure increased the interaction of SIRT2 (a deacetylase) with PFKP (a glycolytic enzyme), leading to PFKP deacetylation and degradation via ubiquitination. This SIRT2-PFKP crosstalk affected phagocytosis and LAP activation by reducing PFKP levels and impairing Atg4B-LC3-mediated LAP activation. Atg4B is a cysteine protease that binds to LC3 and facilitates autophagosome formation. Inhibiting SIRT2 restored LAP activity and normalized phagocytosis in sepsis conditions (64). According to Samantha et al., alcohol exposure increased the expression of C/EBPβ, which negatively affected the phagocytosis function and the PPAR-γ pathway in alveolar macrophages. However, when C/EBPβ was inhibited by the PPAR-γ ligand, pioglitazone, the phagocytic activity was restored (65). Therefore, these pathways could be potential targets for the treatment of diseases related to impaired efferocytosis. Insufficient studies have been conducted to establish a direct correlation between alcohol-induced efferocytosis dysfunction and the precise pathway of disease exacerbation. Further investigation is imperative to elucidate the definitive mechanisms of pathology associated with alcohol consumption.

## Possible therapeutic targets to promote efferocytosis

Contemporary research is increasingly focused on elucidating the mechanisms of efferocytosis impairment and its pathological significance in various diseases. It has been demonstrated that anomalies in the ‘find me’ and ‘eat me’ signals, coupled with disruptions in the internalization phase, significantly affect efferocytosis efficiency. Phagocytes, primarily responsible for efferocytosis, are adversely affected by the deficiency or loss of Tyro, Axl, and MerTK (TAM) receptors. Furthermore, the ADAM family of proteins is instrumental in the proteolytic shedding of TAM receptors, a process critical to efferocytosis. Modulating TAM receptor activity or inhibiting ADAM17 has been shown to enhance efferocytosis, offering therapeutic potential in disease states such as ALI and RA (52, 66). Overexpression of ‘don’t eat me’ signals, such as CD47, imparts self-tolerance in apoptotic cells, modulating their recognition and subsequent phagocytosis. Preclinical models suggest that statins, capable of inhibiting CD47 and RhoA activation, may be efficacious (67). Additionally, targeting pathways such as AMPK, PPAR-γ, and LXR, which promote Rac1 activation, anti-inflammatory cytokine production, and apoptotic cell recognition, could enhance the consistent removal of dead cells (68). Metformin, an AMPK agonist, emerges as a novel approach to ameliorate efferocytosis deficits. Research indicates that overexpression of ADAM9 and reduced miR-126 expression diminish efferocytosis in diabetic conditions, whereas inhibiting ADAM9 or augmenting miR-126 expression can restore efferocytosis (69). Thus, interventions at the molecular level may offer significant therapeutic advantages in reversing impaired efferocytosis. Accumulating evidence indicates that a deficiency in MFG-E8 is associated with the exacerbation of autoimmune conditions. Furthermore, alcohol intake has been observed to significantly reduce MFG-E8 levels while concurrently increasing HMGB1 production, a type of damage-associated molecular pattern (DAMP) (52). Regarding alcohol-mediated efferocytosis impairment, the research posits that attenuating HMGB1 may facilitate the restoration of efferocytosis. Enhancing our understanding of the regulatory mechanisms of efferocytosis and identifying modalities to stimulate this process could offer promising therapeutic strategies for alcoholism and other inflammatory diseases. To date, the majority of efferocytosis research has been confined to cellular and animal models, with minimal advancement in clinical applications. There is an imperative need for further studies to investigate the effects of efferocytosis modulation on the predisposition to inflammatory diseases and to evaluate the clinical safety of such interventions.

## Conclusion

The phagocytic clearance of apoptotic cells is a key mechanism for the host system to maintain cellular homeostasis and self-tolerance. However, alcohol exposure can induce genetic aberration or immunosuppression that activates the inflammatory signaling pathway. A recent study showed that the defective communication between the apoptotic cell and the phagocyte impairs efferocytosis and contributes to various inflammatory diseases. This review discusses how acute and chronic alcohol exposure triggers inflammatory responses and efferocytosis dysfunction that leads to adverse disease outcomes. Moreover, it explores the novel approach of targeting the efferocytosis process to prevent or treat alcohol-related severe inflammatory diseases.

## Author contributions

SB: Conceptualization, Writing – original draft, Writing – review & editing. RT: Conceptualization, Investigation, Supervision, Writing – review & editing.
